# The British Medical Association and Their Midwives Bills

**Published:** 1896-07-04

**Authors:** 


### The British Medical Association and their Midwives Bills.

A meeting of the Metropolitan Branch of the British Medical Association was held on June 26th "to consider the question of the registration of midwives," and to frame a reply to a letter from the general secretary on the subject of certain Bills for the registration of midwifery nurses.

After attention had been called to various less interesting matters, the fact was pointed out that, while the meeting was called to consider the question of the registration of midwives, the two Bills which were to be discussed were entirely on the subject of midwifery nurses. To some it might—and, in fact, it did—seem that this was a trifle over which it was hardly worth while wasting the time of the meeting. A glance, however, at those by whom this point was raised showed that it came from very opposite quarters, and it was not long before the admission of the chairman—which also seemed to meet with the general approval of the meeting—that really this was but a small difference, was taken hold of in a somewhat amusing manner, for on the clause being reached in which the short title is given as the Midwifery Nurses Act, an amendment to substitute the word midwives for the term midwifery nurses was urged, if one may so say, from both sides of the House, and clearly for the most opposite of reasons. The advocates of the midwives have all along objected to the term midwifery nurses as being without meaning and inapplicable to existing midwives, so they were glad enough to remove this obnoxious appellation from the Bill. But those who objected *in toto* to midwives and all their ways seemed equally desirous of seeing the old word midwife taking the place of the new-fangled title, midwifery nurse, Dr. Bedford Fenwick, in fact, calling for the change in the name of honesty, for, as he said, whatever the wording of the Bill, those who were responsible for it when they spoke of midwifery nurses really meant midwives. So the advocates of the midwives were supported by those who usually oppose them as well as by those who thought the name a matter of indifference, and all this strange company seemed on the point of voting that midwives it should be. But there was in all this an *arrière pensée*. The British Medical Association, however pleasantly it may flirt with the idea of midwifery nurses, has already in public *séance* protested against the whole scheme of registering midwives, a scheme which in the records of the Association stands condemned, lock, stock, and barrel. The only effect, then, of the almost success of those who love the name of "midwife" to get rid of the compound title was to land the Bill in the *index prohibitorius*, for had not the Association already spoken in regard to midwives? It was then a barren victory, for such logical faculty as was left to the meeting after these argumentative feats made it easy to impress upon them that if a midwifery nurse is equal to a midwife, and if from an Association point of view a midwife is taboo, a midwifery nurse is taboo also; and thus it was not difficult for Dr. Hugh Woods to carry his resolution to the effect that the meeting could not approve of either of the Bills. The real question, however, as to what is to be done to improve the education of the midwives, who admittedly attend more than half the confinements in the country, was but slightly touched upon. Still some few of those present did seem to see that all this objection to recognising or legislating for midwives does not in any way alter the troublesome fact that they exist and occupy the field, and that their proceedings ought to be regulated in some way or another. One speaker went so far as to say that, much as the public deals with the social evil, shutting its eyes to it, notwithstanding the publicity of its ways, so we have got into the way of pretending to ourselves and acting as if the midwife did not exist, although we know very well that she does exist all the time. The simile was not very polite, perhaps, but it was an accurate enough description of the mental condition of many who discuss this question. It is all very well to oppose legislation. But what is to be done to help and protect the thousands of poor women who are now attended by midwives confessedly ignorant and untrained?

